# Demographic Factors, Conspiracy Theories, and Beliefs Associated With COVID-19 Vaccine Hesitancy Among Pakistani Population: A Cross-Sectional Study

**DOI:** 10.7759/cureus.32629

**Published:** 2022-12-17

**Authors:** Aabiya Arif, Sameer S Tebha, Arfa Badar, Mohammad Aadil Qamar, Rubaid A Dhillon, Syed Akbar Abbas, Minahil Tariq

**Affiliations:** 1 Medicine, Ziauddin Medical College, Karachi, PAK; 2 Neurosurgery and Neurology, Jinnah Medical & Dental College, Karachi, PAK; 3 Clinical and Translational Research, Larkin Community Hospital, Miami, USA; 4 Computer Science and Information Technology, Institute of Business Management, Karachi, PAK; 5 Internal Medicine, Riphah International University, Rawalpindi, PAK; 6 Otolaryngology - Head and Neck Surgery, Aga Khan University Hospital, Karachi, PAK; 7 Medical College, Liaquat College of Medicine & Dentistry, Karachi, PAK

**Keywords:** vaccine conspiracy belief scale, conspiracy mentality questionnaire, kuppuswamy socioeconomic scale, vaccination campaign, conspiracy theories, pakistan, vaccine hesitancy, covid-19 vaccine, sars-cov-2 infection, covid-19

## Abstract

Introduction

The coverage of coronavirus disease 2019 (COVID-19) immunization uptake has been impacted by the acceptance of regular vaccine uptake and, like many other vaccination attempts in the past, it also faces issues of vaccine hesitancy. Through this study, we hoped to assess the conspiracy theories and beliefs attached to the COVID-19 vaccination efforts in Pakistan

Methods

This study was conducted among the general population of Pakistan, aged 16 and above, from April 29 to May 29, 2021. The data was collected through English and Urdu questionnaires by a method of convenient sampling. A total of 600 participants were asked to fill in 34 questions pertaining to vaccine hesitancy and conspiracies. We used the Modified Kuppuswamy Socioeconomic Scale, Kuppuswamy Socioeconomic Scale, Conspiracy Mentality Questionnaire (CMQ), and Vaccine Conspiracy Belief Scale (VCBS) as our questions.

Results

A total of 591 participants responded to our questionnaire. The majority were females (56.7%), had an undergraduate degree (41.5%), and belonged to the upper middle socioeconomic class (40%). Factors like occupation (*p=*0.001), education (*p=*0.001), socioeconomic status (*p<*0.001), any family member who contracted coronavirus (*p=*0.016), source of knowledge (*p=*0.002), and total monthly income (*p<*0.001) were found to be statistically significant.

Conclusion

The findings of this study suggest that conspiracy theories and misinformation about vaccinations are prevalent in our region. They are influenced by propaganda and negative cultural values among the population To combat and restrict the spread of this problem, our study can provide useful data to develop more appropriate policy measures.

## Introduction

Severe acute respiratory syndrome coronavirus 2 (SARS-CoV-2) has wreaked havoc on the world's humanitarian and economic systems and has infected more than 648 million, killing approximately 6.6 million human beings worldwide as of December 2022 [1]. Throughout its course, a lot of effort and resources were attached to developing an effective vaccine against this virus. However, the availability of vaccines against coronavirus disease 2019 (COVID-19), that too in record time, lead to another struggle for health professionals, similar to previous experiences with vaccine hesitancy among people.

Vaccine hesitancy, defined as the refusal of vaccination despite the availability of vaccination services [2], has burdened immunization practice long before the ongoing COVID-19 pandemic. Immunization has lowered the burden of infectious diseases and their related morbidity and mortality and has increased average life expectancy [3]. Through preventive interventions by vaccination, 2.5-3 million lives are saved each year. According to some estimates, another 1.5 million could be added to this number if worldwide vaccination efforts are enhanced [4]. However, despite such compelling evidence favoring vaccines, global vaccine uptake rates are substandard [5]; in 2019 alone, World Health Organization (WHO) added vaccine hesitancy to the top 10 global health threats.

Vaccine hesitancy remains a significant concern for Pakistan. The country has the highest number of unvaccinated children in the world12. Despite the Expanded Program on Immunization's efforts since 1978, the national immunization rate has remained comparatively low when in comparison to other regional nations like India and Bangladesh, with two-thirds of youngsters receiving complete vaccinations [5-6]. Furthermore, vaccine-preventable diseases account for 50% of infant fatalities in Pakistan, the world's highest mortality rate, allowing for persistently poor vaccination rates [5]. Vaccine hesitancy has a significant contribution to the development of these statistics.

Moreover, low literacy rates, socioeconomic disparities, lack of access to information in rural regions owing to a lack of technology, and religious and cultural beliefs are all factors that contribute to vaccine hesitancy. However, conspiracy theories play a more significant role in Pakistan [7-9]. Unfortunately, since the early days of this pandemic, multiple conspiracy theories have spread uncontrollably through social media in Pakistan. For example, the virus is a magnificent deception, or the COVID-19 vaccine will be used as an excuse to deliver nanochips to seize control [7]. Misconceptions regarding the aim or effectiveness of immunization, such as vaccines can harm or sterilize the person, or theories claiming the use of a low-quality vaccine or a vaccine containing monkey- or swine-derived compounds may have cultural and religious sensitivity leading to resistance. These factors have also significantly affected Pakistan's failure to eradicate polio [6,7,9].

In addition, the tendency for the disease to spread in Pakistan is high due to a weak healthcare system, dense population, and lack of compliance with sanitation regulations, rendering Pakistan extremely vulnerable to emerging, more transmissible COVID-19 variants [8]. With the failure to fully implement standard operating procedures (SOPs) and due to the country's economic situation, long-term lockdown, and travel ban not being a first-line defense measure to combat the pandemic, immunization is the only way to safeguard Pakistan from future catastrophic waves [7,8]. However, the effectiveness of vaccination programs depends on high vaccine acceptance. Accordingly, the success of these programs might be jeopardized by the propagation of conspiracies [8].

Hence, this study aims to identify the factors linked with vaccine hesitancy and discover the common misconceptions regarding vaccines in general, and COVID-19 vaccines, among Pakistan's general population. The findings of this study shall aid in making more targeted efforts towards vaccine hesitancy amid COVID-19 for better outcomes.

## Materials and methods

The data used in this observational study were collected using an online questionnaire in English and Urdu, between April 29 and May 29, 2021. The general population of Pakistan, aged 16 and above, was the target demographic for this study. A total of 34 questions were included in the final questionnaire. Divided into four sections, the questionnaire was validated through a pilot study that included 20 participants, the results of which are not part of the present sample. The English version of our questionnaire can be found in the appendix. Social media platforms such as Facebook, Instagram, and WhatsApp (Meta Platforms, Inc., Menlo Park, California, United States) were used to collect responses for the English questionnaire. The Urdu questionnaires were physically distributed, mainly in areas with little to no use of social media and low literacy rates and English literacy; these included parts of North and Eastern Karachi and the suburbs of the city. Both questionnaires were self administered. Moreover, assistance was provided to those who could not read either of the languages. The sample size of 385 with a 95% confidence level (CI) was calculated using the tool OpenEpi (Open Source Epidemiologic Statistics for Public Health), which used the formula n=[DEFF*Np(1-p)]/[(d2/Z21-a/2*(N-1)+p*(1-p)] and was increased by 56% to 600 participants since we were able to get more responses [10]. Nine responses were incomplete; hence, a total of 591 responses were collected. 

The study was approved by the Ethical Committee of Islamic International Medical College (Approval number: Riphah/IIMC/ ERC/184). Participation in the study was entirely voluntary, and all collected data were treated with confidentiality. An informed consent form was included in the online survey, and consent was verbally obtained in the case of the Urdu questionnaire. 

The modified Kuppuswamy Socioeconomic Scale consists of three questions: education and occupation of the family head, and income per month. This yields a score of 3-29. This scale divides the socioeconomic classes into five, where a score of 3 refers to the lower class and 29 to the upper class [11]. Along with the Kuppuswamy Socioeconomic Scale, demographic questions and a history of chronic disease or COVID-19 infection made up the first section.

The second section included questions regarding conspiracy theories about COVID-19's origin and knowledge and attitudes about COVID-19 vaccines. Finally, the third section was dedicated to the Conspiracy Mentality Questionnaire (CMQ), a five-item questionnaire meant to test a person's propensity towards conspiracy theories in general (not vaccine-related) [12]. Participants were asked to rate how much they agreed or disagreed with each of the five statements on a five-point Likert scale, ranging from "strongly disagree," which received a minimum score of 1, to "strongly agree," which received a maximum score of 5. CMQ was tested in a broad cross-cultural population and is consistent over time. 

The fourth section comprised the Vaccine Conspiracy Belief Scale (VCBS), validated previously [13]. This scale includes seven statements, and the participants were asked to rate how much they agreed or disagreed with each of the seven statements on a seven-point scale. The scale ranged from "strongly disagree," which received a score of one, to "strongly agree," which received a score of seven. Greater belief in vaccine conspiracies is associated with higher VCBS scores. 

IBM SPSS Statistics for Windows, Version 21.0 (IBM Corp., Armonk, New York, United States) was used for statistical analysis. To examine the association between variables, the chi-squared (χ2) test, Mann-Whitney U test, and the Kruskal-Wallis (K-W) test were utilized where appropriate. A p-value of <0.05 was considered statistically significant, and the CI was set at 95%. 

## Results

Participant demographics and COVID-19 vaccine acceptance 

A total of 591 adults responded to the study. Most study participants were females (56.7%), were aged between 18-25-year-old (65.0%), single (69.2%), had an undergraduate education level (41.5%), and belonged to the upper-middle socioeconomic class (40%) (Table 1). Upon the question of COVID-19 vaccine acceptance, 487 (82.4%) participants showed their willingness. Statistically significant factors amongst participants' demographics that had an association with vaccine acceptance were the occupation of the participants (p=0.001), education of the participants (p=0.001), socioeconomic status of the participants (p<0.001), any family member who contracted coronavirus (p=0.016), source of knowledge (p=0.002), and total monthly income (p<0.001) (Table 1). 

**Table 1:** Demographical data and coronavirus vaccine acceptance *p-value <0.05 statistically significant COVID-19: coronavirus disease 2019

Variable		Total		COVID-19 Vaccine Acceptance				p-value (Chi-square test)
				Yes (n=487)			No (n=104)	
Gender	Male	256 (43.3%)			211 (82.4%)	45 (17.6%)		0.991	
	Female	335 (56.7%)			276 (82.4%)	59 (17.6%)			
Age Group	18-25	384 (65.0%)			311 (81.0%)	73 (19.0%)		0.058	
	26-40	124 (21.0%)			103 (83.1%)	21 (16.9%)			
	41-60	72 (12.2%)			66 (91.7%)	6 (8.3%)			
	≥61	11 (1.9%)			7 (63.6%)	4 (36.4%)			
Marital Status	Single	409 (69.2%)			337 (69.2%)	72 (69.2%)		0.179	
	Married	168 (28.4%)			141 (29.0%)	27 (26.0%)			
	Separated/divorced/widow	14 (2.4%)			9 (1.8%)	5 (4.8%)			
Province	Sindh	463 (78.3%)			377 (81.4%)	86 (18.6%)		0.235	
	Others	128 (21.7%)			110 (85.9%)	18 (14.1%)			
Occupation	Medical student	154 (26.1%)			134 (87.0%)	20 (13.0%)		0.001*	
	Student	173 (29.3%)			136 (78.6%)	37 (21.4%)			
	Healthcare worker	48 (8.1%)			39 (81.3%)	9 (18.8%)			
	Private sector	119 (20.1%)			109 (91.6%)	10 (8.4%)			
	Housewife	44 (7.4%)			34 (77.3%)	10 (22.7%)			
	Government job	9 (1.5%)			7 (77.8%)	2 (22.2%)			
	Household workers	44 (7.4%)			28 (63.6%)	16 (36.4%)			
Education	Uneducated	26 (4.4%)			16 (61.5%)	10 (38.5%)		0.001*	
	Less than matriculation	12 (2%)			7 (58.3%)	5 (41.7%)			
	Matriculation/ O-Level	74 (12.5%)			55 (74.3%)	19 (25.7%)			
	Inter/ A-Level	87 (14.7%)			74 (85.1%)	13 (14.9%)			
	Undergraduate	245 (41.5%)			204 (83.3%)	41 (16.7%)			
	Postgraduate	147 (24.9%)			131 (89.1%)	16 (10.9%)			
Socioeconomic Status	Upper	229 (39%)			199 (86.9%)	30 (13.1%)		<0.001*	
	Upper middle	237 (40%)			200 (84.4%)	37 (15.6%)			
	Lower middle	51 (9%)			40 (78.4%)	11 (21.6%)			
	Upper lower	66 (11%)			45 (68.2%)	21 (31.8%)			
	Lower	8 (1%)			3 (37.5%)	5 (62.5%)			
Chronic Disease	Yes	72 (12%)			61 (84.7%)	11 (15.3%)		0.581	
	No	519 (88%)			426 (82%)	93 (17.9%)			
Family member contraction with coronavirus	Yes	196 (33%)			174 (88.8%)	22 (11.2%)		0.016*	
	No	347 (59%)			275 (79.3%)	72 (20.7%)			
	May be	48 (8%)			38 (79.2%)	10 (20.8%)			
Source of Knowledge	Healthcare professionals	118 (20%)			109 (92.4%)	9 (7.6%)		0.002*	
	Social media	284 (48%)			230 (81.0%)	54 (19.0%)			
	Family members/friends	48 (8%)			32 (66.7%)	16 (33.3%)			
	Newspaper	26 (4%)			22 (84.6%)	4 (15.4%)			
	Television	115 (19%)			94 (81.7%)	21 (18.3%)			
Total Monthly Income	≥ 199,862	171 (28.9%)			150 (87.7%)	21 (12.3%)		<0.001*	
	99,931–199,861	141 (23.9%)			125 (88.7%)	16 (11.3%)			

Conspiracy mentality 

**Figure 1:**
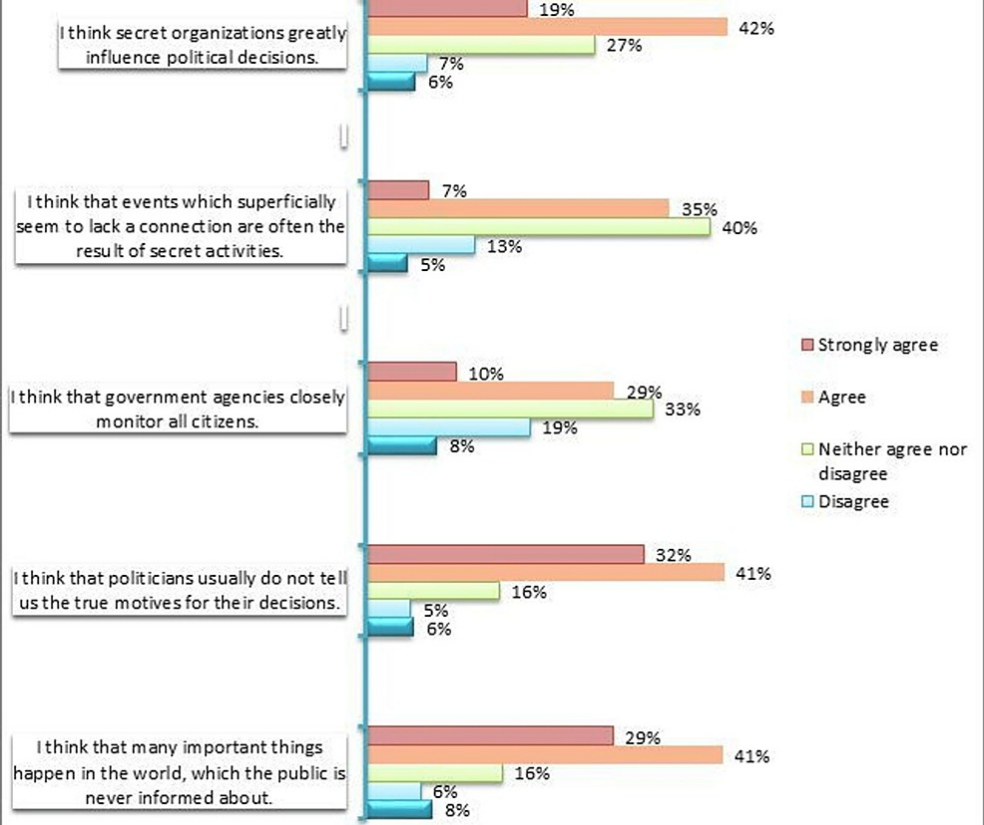
Frequency of various responses from the conspiracy mentality questionnaire (n=591)

Study participants were assessed using the CMQ. The majority of the participants agreed with statements such as, 'I think secret organizations greatly influence political decisions,' 'I think that politicians usually do not tell us the true motives for their decisions,' and 'I think that many important things happen in the world, which the public is never informed about' accounting to 42%, 41%, and 41%, respectively (Figure 1). Our participants demonstrated neutrality when asked to comment on the statements, 'I think that events which superficially seem to lack a connection are often the result of secret activities,' and 'I think that government agencies closely monitor all citizens' accounting for 40% and 33%, respectively (Figure 1). 

The sub-group analysis of demographical variables showed a statistically significant difference amongst the mean scores of different age categories (p<0.001), with the highest score in the age group of 18-25 years (66.7±17.3), marital status (<0.001) where being single showed a higher score (66.3±17.7), province (p=0.017) with provinces other than Sindh reported greater score (67.7±17.3), occupation (p<0.001), where being a medical student (68.7±15.6) corresponded to the highest score, education (p<0.001) where having studied till Inter/A-Level (70.2±13.2) was the highest-scoring category, monthly income (p<0.001) where the highest scoring group was the Rs.99,931-199,861 (69.0±15.4), socioeconomic status (p<0.001) with the upper class (67.2±16.8) reporting the greatest score, having a family member who has/had contracted coronavirus (66.6±16.7, p<0.001) and source of knowledge (p=0.028) with healthcare professionals (67.0±17.9) scoring the highest score (Table 2). 

**Table 2:** Mean comparison for conspiracy mentality questionnaire *p-value <0.05 statistically significant SD: Standard deviation, MW: Mann-Whitney test, KW: Kruskal-Wallis test

Variable		Mean ±. S.D.	P-value
Gender	Male	62.29 ± 21.45	0.217 MW
	Female	64.27 ± 18.59	
Age Category	18-25	66.7 ± 17.3	<0.001* KW
	26-40	60.9 ± 22.5	
	41-60	53.1 ± 22.1	
	≥61	43.6 ± 22.3	
Marital Status	Single	66.3 ± 17.7	<0.001*
	Married	59.2 ± 21.2	
	Separated/widow/widower	28.9 ± 24.9	
Province	Sindh	62.2 ± 20.4	0.017*
	Other provinces	67.7 ± 17.3	
Occupation	Medical student	68.7 ± 15.6	<0.001*
	Student	66.3 ± 16.6	
	Healthcare worker	63.1 ± 22.4	
	Private sector	63.5 ± 19.5	
	Homemaker/housewife	54 ± 24.8	
	Government job	68.3 ± 14.1	
	Household workers (maid, driver)	42 ± 22.8	
Education	Uneducated	33.1 ± 22.5	<0.001*
	Less than matriculation/O-Level	34.6 ± 18.5	
	Matriculation/O-Level	59.1 ± 23.1	
	Inter/A-Level	70.2 ± 13.2	
	Undergraduate	67.1 ± 16.7	
	Postgraduate	63.1 ± 18.9	
Monthly Income	≥ 199,862	65.4 ± 18.4	<0.001*
	99,931–199,861	69 ± 15.4	
	74,755 –99,930	67.3 ± 15.7	
	49,962–74,755	66.8 ± 15.8	
	29,973– 49,961	58.9 ± 22.3	
	10,002–29,972	43.6 ± 21.3	
	≤ 10,001	49.6 ± 29.5	
Socioeconomic Status	Upper	67.2 ± 16.8	<0.001*
	Upper middle	66.5 ± 17.9	
	Lower middle	61.2 ± 16.8	
	Upper lower	43.9 ± 24.1	
	Lower	38.1 ± 26.4	
Chronic Disease	Yes	60.3 ± 19.7	0.075 MW
	No	63.8 ± 19.9	
Family member contraction with coronavirus	Yes	66.6 ± 16.7	<0.001*
	May be	70.5 ± 16.6	
	No	63.4 ± 19.9	
Source of Knowledge	Healthcare professionals	67 ± 17.9	0.028*
	Social media	64.2 ± 19.6	
	Family members/friends	58.6 ± 21.7	
	Newspaper	56 ± 19.7	
	Television	61.3 ± 21.1	

Belief in Covid-19 vaccine conspiracies 

**Figure 2:**
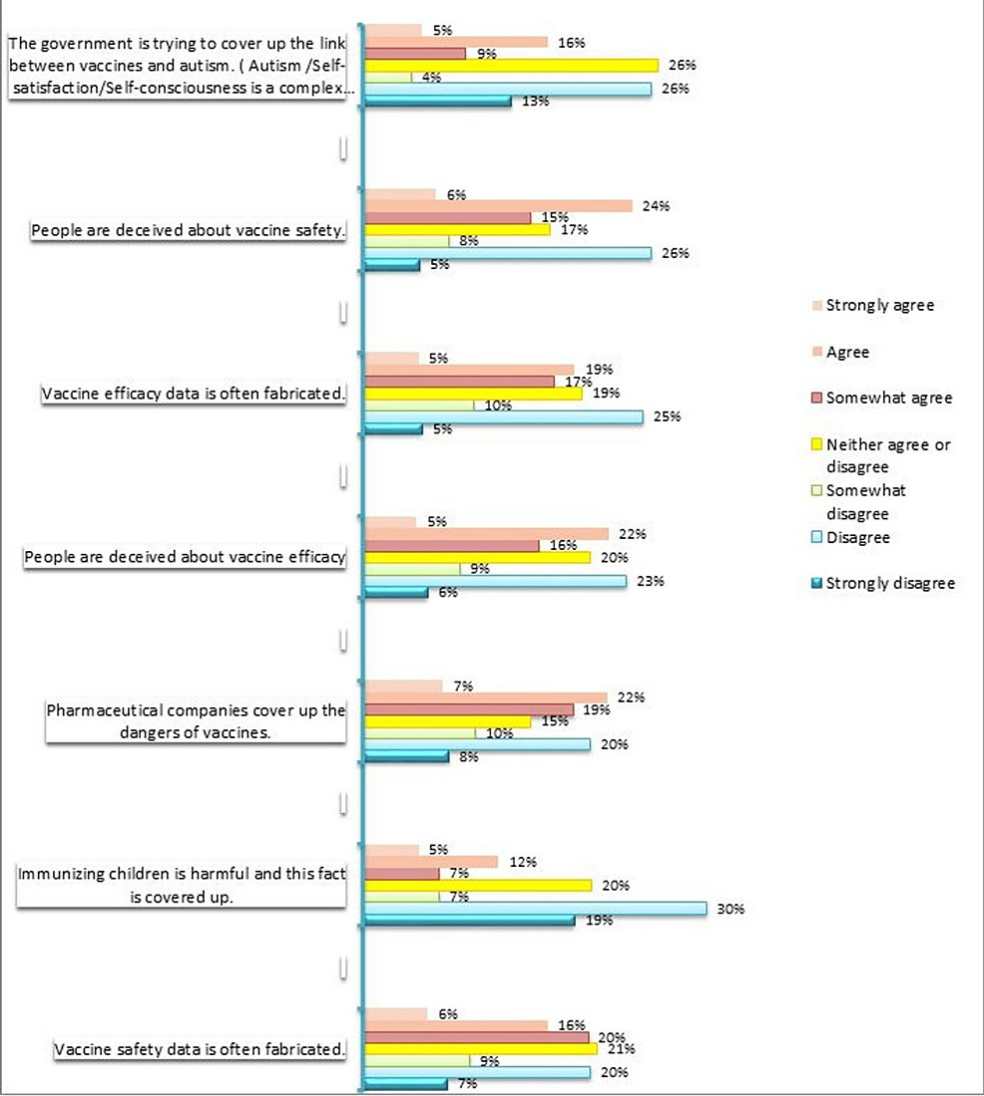
Frequency of various responses from the vaccine conspiracy belief scale (n=591)

Participants were assessed for their beliefs regarding conspiracy theories via the VCBS. Overall, 30%, 26%, 25%, and 23% of the respondents disagreed with the statement 'Immunizing children is harmful, and this fact is covered up,' 'People are deceived about vaccine safety,' Vaccine efficacy data are often fabricated,' and 'People are deceived about vaccine efficacy' (Figure 2). Furthermore, 22% of the respondents agreed that 'Pharmaceutical companies cover up the dangers of vaccines,' with 21% being neutral to the statement 'Vaccine safety data is often fabricated' (Figure 2). 

Upon comparing the means of different sub-groups within demographical variables, a statistically significant difference was found in different occupations (p=0.014), with the homemaker/ housewife scoring the highest score, monthly income (p=0.001), where participants with a monthly income of Rs. ≤10,001 (60.2±26.5) scored the highest score, socioeconomic status (p=0.010) with the lower class (59.5±30.8) scoring the greatest, presence of chronic disease (41.4±24.4, p=0.010), and the source of knowledge (p<0.001) where using social media (50.7±21.2) as the source reported the highest score (Table 3). 

**Table 3:** Mean comparison for vaccine conspiracy belief scale *p-value <0.05 statistically significant MW: Man-Whitney; KW: Kruskal Wallis; SD: standard deviation

Variable		Mean ± S.D.	p-value
Gender	Male	47.4 ± 22	0.979 MW
	Female	47.6 ± 22.5	
Age Category	18-25	47.6 ± 21.7	0.134 KW
	26-40	49.8 ± 24.1	
	41-60	42.6 ± 21.7	
	≥61	50.9 ± 24.1	
Marital Status	Single	46.8 ± 21.7	0.465
	Married	49.2 ± 22.9	
	Separated/widow/widower	47.1 ± 29.7	
Province	Sindh	47.7 ± 22.4	0.628
	other provinces	46.9 ± 21.9	
Occupation	Medical student	42 ± 22.6	0.014*
	Student	50.2 ± 20.1	
	Healthcare worker	49.1 ± 26.2	
	Private sector	48.9 ± 22.2	
	Homemaker/housewife	53.9 ± 20	
	Government job	40.7 ± 28.2	
	Household workers (maid, driver)	45.6 ± 22.7	
Education	Uneducated	48.5 ± 25.8	0.463
	Less than matriculation/O-Level	51.4 ± 22.1	
	Matriculation/O-Level	48.8 ± 23.3	
	Inter/A-Level	48.6 ± 19.9	
	Undergraduate	45 ± 22	
	Postgraduate	49.8 ± 22.9	
Monthly Income	≥ 199,862	41.4 ± 22.8	0.001*
	99,931–199,861	48.5 ± 22.1	
	74,755 –99,930	51.1 ± 19.3	
	49,962–74,755	50.1 ± 22	
	29,973– 49,961	47 ± 23.2	
	10,002–29,972	50.2 ± 17.2	
	≤ 10,001	60.2 ± 26.5	
Socioeconomic Status	Upper	43.5 ± 23.6	0.010*
	Upper middle	50 ± 20.3	
	Lower middle	50.3 ± 21.8	
	Upper lower	48.9 ± 21.8	
	Lower	59.5 ± 30.8	
Chronic Disease	Yes	41.4 ± 24.4	0.010*
	No	48.4 ± 21.9	
Family member contraction with coronavirus	Yes	47.7 ± 21.7	0.955
	May be	47.9 ± 23.9	
	No	47.3 ± 22.4	
Source of Knowledge	Healthcare professionals	38.9 ± 24.6	<0.001*
	Social media	50.7 ± 21.2	
	Family members/friends	47.9 ± 18.3	
	Newspaper	43.4 ± 25.4	
	Television	49.1 ± 21.1	

Univariate and multivariate analysis of COVID-19 vaccine acceptance 

Univariate analysis of occupations showed that being a medical student (OR: 3.83, 95%CI: 1.77-8.30, p=0.001), student (OR: 2.10, 95%CI: 1.03-4.29, p=0.042), and working in the private sector (OR: 6.23, 95%CI: 2.55-15.21, p<0.001) were significantly associated with vaccine acceptance (Table 4). In the educational categories, having an undergraduate (OR: 3.11, 95%CI: 1.32-7.34, p=0.012), and postgraduate (OR: 5.12, 95%CI: 1.99-13.17, p=0.010) level of education was associated with increased odds of vaccine acceptance (Table 4). Our analysis reported decreased odds of vaccine acceptance in participants with a monthly income of Rs. 10,002-29,972 (OR: 0.14, 95%CI: 0.06-0.33, p<0.001), Rs. 29,973-49,961 (OR: 0.35, 95%CI: 0.16-0.76, p=0.008), and Rs. 49,962-74,755 (OR: 0.37, 95%CI: 0.18-0.76, p=0.007) (Table 4). 

**Table 4:** Univariate and multivariate analysis of demographical data, conspiracy mentality questionnaire, and vaccine conspiracy belief scale *p-value <0.05 statistically significant COVID-19: coronavirus disease 2019; CI: confidence interval

Variables	Univariate analysis						Multivariable analysis		
	OR (95% CI)					p-value	OR (95% CI)	p-value	
Gender									
Female	Reference								
Male	0.998 (0.651-4.530)					0.991			
Age	1.004 (0.986-1.023)					0.657			
Occupation									
Household workers	Reference								
Medical student	3.83 (1.77-8.3)				0.001*		1.281 (0.308-5.323)	0.733	
Student	2.1 (1.03-4.29)				0.042*		1.229 (0.336-4.5)	0.755	
Healthcare worker	2.48 (0.96-6.4)				0.061		1.055 (0.209-5.311)	0.949	
Private sector	6.23 (2.55-15.21)				0.000*		2.905 (0.667-12.648)	0.155	
Housewife	1.94 (0.76-4.95)				0.164		1.946 (0.493-7.687)	0.342	
Government job	2 (0.37-10.81)				0.421		0.966 (0.074-12.543)	0.979	
Education									
Uneducated	Reference								
Less than matriculation	0.875 (0.217-3.52)				0.002*		0.775 (0.106-5.672)		0.802
Matriculation/O-Level	1.809 (0.702-4.66)				0.851		1.104 (0.269-4.525)		0.891
Inter/A-Level	3.558 (1.328-9.53)				0.220		1.507 (0.302-7.517)		0.617
Undergraduate	3.11 (1.318-7.34)				0.012*		1.073 (0.236-4.877)		0.928
Postgraduate	5.117 (1.988-13.17)				0.010*		2.313 (0.464-11.54)		0.306
Monthly income									
≤ 10,001	Reference								
10,002-29,972	0.14 (0.059-0.33)				0.000*		1.647 (0.445-6.095)		0.455
29,973- 49,961	0.35 (0.162-0.76)				0.008*		0.795 (0.215-2.942)		0.731
49,962-74,755	0.371 (0.18-0.76)				0.007*		1.274 (0.305-5.311)		0.740
74,755 -99,930	0.624 (0.281-1.38)				0.246		2.393 (0.575-9.962)		0.230
99,931-199,861	0.878 (0.401-1.92)				0.745		4.659 (1.025-21.18)		0.046*
≥ 199,862	1.094 (0.547-2.19)				0.800		2.633 (0.555-12.483)		0.223
Socioeconomic status									
Upper	Reference								
Upper middle	0.815 (0.484-1.37)				0.440		1.772 (0.672-4.675)		0.248
Lower middle	0.548 (0.254-1.18)				0.126		3.036 (0.775-11.89)		0.111
Upper lower	0.323 (0.17-0.62)				0.001*		1.913 (0.4-9.148)		0.416
Lower	0.09 (0.021-0.4)				0.001*		0.538 (0.05-5.849)		0.611
Family member contraction with COVID-19									
No	Reference								
Yes	2.071 (1.239-3.46)				0.005*		2.122 (1.12-4.023)		0.021*
May be	0.995 (0.473-2.09)				0.989		0.849 (0.345-2.086)		0.720
Source of knowledge									
Healthcare professionals				Reference					
Social media		0.352 (0.168-0.74)			0.006*		0.709 (0.287-1.751)		0.456
Family members/friends		0.165 (0.067-0.41)			0.000*		0.246 (0.078-0.782)		0.017*
Newspaper		0.454 (0.128-1.61)			0.221		0.565 (0.101-3.159)		0.516
Television		0.37 (0.161-0.85)			0.018*		0.691 (0.246-1.944)		0.484
Belief about the origin of COVID-19									
Natural	Reference								
Artificial	0.394 (0.255-0.607)				0.000*		0.678 (0.378-1.216)		0.192
Believe that the coronavirus was artificial to force everyone to get vaccinated.									
No	Reference								
Yes	0.319 (0.206-0.492)				0.000*		0.855 (0.453-1.613)		0.629
Believe that vaccines will be a way of implanting people with microchips to control humans.									
No	Reference								
Yes	0.216 (0.134-0.350)				0.000*		0.695 (0.348-1.39)		0.304
Believe that COVID-19 vaccines will lead to infertility?									
No	Reference								
Yes	0.160 (0.098-0.259)				0.000*		0.43 (0.218-0.85)		0.015*
Conspiracy mentality	1.002 (0.992-1.013)				0.705				
Vaccine conspiracy Belief			0.953 (0.941-0.965)		0.000*		0.957 (0.943-0.972)		0.000*

Stratification of the socioeconomic status of the participants showed that the upper-lower class (OR: 0.32, 95%CI: 0.17-0.62, p=0.001), and lower class (OR: 0.09, 95%CI: 0.02-0.40, p=0.001) are more prone to decline vaccination (Table 4). The odds of vaccination were observed to be increased in participants with a family member who had COVID-19 (OR: 2.07, 95%CI: 1.24-3.46, p=0.005). Furthermore, social media (OR: 0.35, 95%CI: 0.17-0.74, p=0.006), family members/ friends (OR: 0.17, 95%CI: 0.07-0.41, p<0.001), and television (OR: 0.37, 95%CI: 0.16-0.85, p=0.018) as a source of knowledge was found to decrease the chances of vaccination in a participant (Table 4). Moreover, the belief about the origin of coronavirus being artificial (OR: 0.39, 95%CI: 0.26-0.61, p<0.001) decreased the odds of vaccination in the participants (Table 4). 

The thought that the coronavirus was artificial to force everyone to get vaccinated (OR: 0.32, 95%CI:0.21-0.49, p<0.001) decreased the odds of a participant getting vaccinated (Table 4). Furthermore, the odds of getting vaccinated were lower in participants who believed that the vaccine would be a way of implanting people with microchips to control humans (OR: 0.22, 95%CI: 0.13-0.35, p<0.001) and thought that COVID-19 vaccines would lead to infertility (OR: 0.16, 95%CI: 0.10-0.25, p<0.001) (Table 4). Further significant findings can be found in Table 4. 

Multivariate analysis showed higher odds of vaccination in participants with a family member who has contacted COVID-19 (OR: 2.122, p=0.021) and monthly income of Rs. 99,931-199,861 (OR: 4.659, p=0.046) while significantly lower odds of vaccination were found in participants who chose family member/friends (OR:0.246, p=0.017) as their source of knowledge and believed that COVID-19 vaccines would lead to infertility (OR: 0.43, p=0.015) (Table 4). 

## Discussion

Among 591 participants that responded to our study proforma, a staggering 82.4% of participants were willing to get vaccinated against COVID-19; among our cohort, the majority of respondents were females between the ages of 18-25 years (65%), had an undergraduate degree (41.5%), and belonged to an upper-middle socioeconomic status (40%). Two previous studies [14,15] have reported vaccine hesitance rates of 31% and 38.8%, respectively, in Bangladesh, which is considerably higher than our study despite the fact that Bangladesh and Pakistan are both culturally, religiously, and ethnically similar countries with a shared colonial past. Nevertheless, we cannot rule out participant selection bias, which is a fundamental limitation of our study. Moreover, data was collected online, and our responses were limited to only one region and one geographic section of Pakistani society, which is highly informed and educated and has ample access to information, considering that a majority had access to social media in our cohort, which could have contributed to a higher vaccine acceptance rate in our respondents. 

Our study shows that participants, although well informed and well educated, still believed in hidden agendas and conspiracy theories, statements like 'I think secret organizations greatly influence political decisions,' 'I think that politicians usually do not tell us the true motives for their decisions,' and 'I think that many important things happen in the world, which the public is never informed about' were accepted by 42%, 41%, and 41%, respectively. Literature has shown that the COVID-19 vaccine has been considered a 'western plot' and in some circles, a 'Jewish conspiracy' to make Muslims impotent; this has more antisemitic roots rather than any reality to it. However, these types of notions are widespread in Muslim-majority Asian nations such as Pakistan, which has, in turn, contributed to the increase in vaccine hesitancy among Muslim-majority Asian nations [8,16]. Moreover, rumors that components such as pork or gelatin are part of the COVID-19 vaccine are also rampant; these substances are prohibited in the Islamic faith and as such, they just further fuel the conspiracy theories and contribute to hesitancy [7,8]. 

Our study showed a direct proportionality with the educational status of the participants, where hesitancy was closely linked with the educational status of the participants. Moreover, hesitancy decreases with an increase in knowledge about the disease. Our study shows that having a trusted source of information like family or friends was statistically significant (p=0.002) for wiliness to get vaccinated. It is in line with evidence published in previous Literature, which agrees that awareness about a disease and the role of vaccination in reducing the mortality and morbidity secondary to that disease reduces vaccine hesitancy among individuals. [17] Moreover, knowledge and awareness regarding the vaccine are significant predictors of hesitancy in studies conducted on COVID-19 and other diseases in low- and middle-income countries such as ours [18-21]. Thus, our study gives ample evidence to drive policy decisions to promote vaccination through targeted awareness campaigns. 

Our cohort participants who had a family member that had contracted COVID-19 previously were highly likely to get vaccinated (p=0.005). This aligns with the human behavior of believing what they can personally experience and relate to, as people are more likely to trust a personal experience rather than believe even undeniable facts and figures. 

The prevalent conspiracy beliefs regarding the COVID-19 vaccine and the hidden role of pharmaceuticals, organizations, agencies, and countries could be playing by planting microchips in humans to control us was one of the leading factors contributing to hesitancy in our study (p=0.000). In multiple studies conducted in low- and middle-income countries, including Pakistan, conspiracy theories have been the culprit leading to vaccine hesitancy and contributed markedly to the limitations of the COVID-19 vaccination program [7]. Moreover, hesitancy toward the vaccine has also been influenced by conspiracy theories in some countries in the Middle East/North Africa (MENA) region [22]. Furthermore, misinformation as to where the virus originated from, regarding vaccine trials, suspicion regarding vaccine manufacturers such as companies or nations [7,22], as well as regarding the efficacy and safety profile have been identified as possible contributing factors in developing countries like Uganda where they further contribute to pre-existing biases leading to further mistrust, confusion, and skepticism over new vaccines, which in turn lowers the wiliness to vaccination [23]. 

This study investigated the pervasiveness and determinants of COVID-19 vaccine hesitancy among the Pakistani population, which could help health policymakers and advisers in developing customized, targeted campaigns to combat vaccine hesitancy in Pakistan as well as in nations that have similar socioeconomic and political circumstances like Pakistan, like the greater MENA and South Asian and Southeast Asian region. However, our study was prone to several limitations considering it was conducted at the peak of COVID-19 time. Firstly, our sample size was smaller and could not include respondents from the rural region of the nation, nor were we able to include participants from diverse communities of the country. The majority of our respondents were from the Urban center of Karachi, Pakistan, although it should be noted all the ethnicities and communities of the countries are represented in the center. Moreover, our sampling technique was convenient sampling; secondly, our data was not representative of the national data in terms of any variable; as such, it can be taken as a representation of some of the population but not all. Lastly, our study employs a cross-sectional study design with its limitation, i.e., we cannot establish causality. 

## Conclusions

Most of our participants were willing to vaccinate themselves. High levels of education and income and having a source of information other than television, family/friends, and social media were all significantly associated with vaccine acceptance. Nevertheless, belief in multiple conspiracy theories and misinformation regarding the vaccine were also prevalent. Conspiracy ideation was significantly more prevalent in young adults, people living in provinces other than Sindh, education up to A levels/intermediate, and the upper socioeconomic class. The findings of this study aid in identifying the group of people more vulnerable to conspiracies, misinformation, and lower vaccine acceptance. Hence, these findings are valuable for assisting in curating targeted strategies to increase vaccine acceptance in Pakistan. 

## Figures and Tables

**Figure 3:**
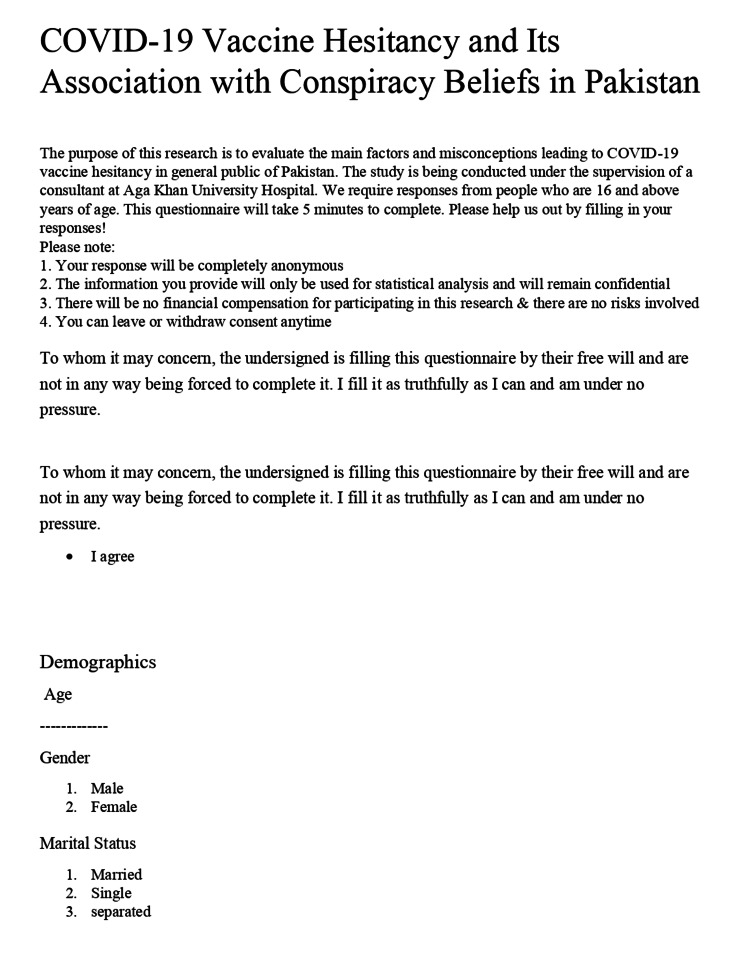
Page 1 of the questionnaire

**Figure 4:**
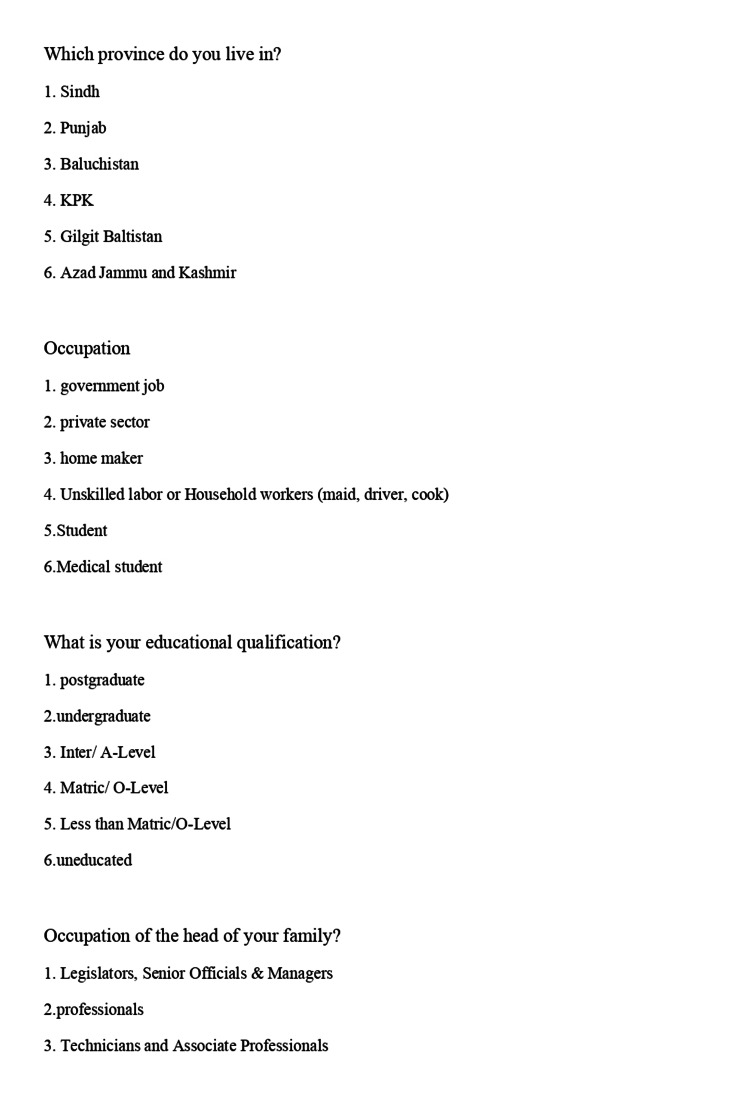
Page 2 of the questionnaire

**Figure 5:**
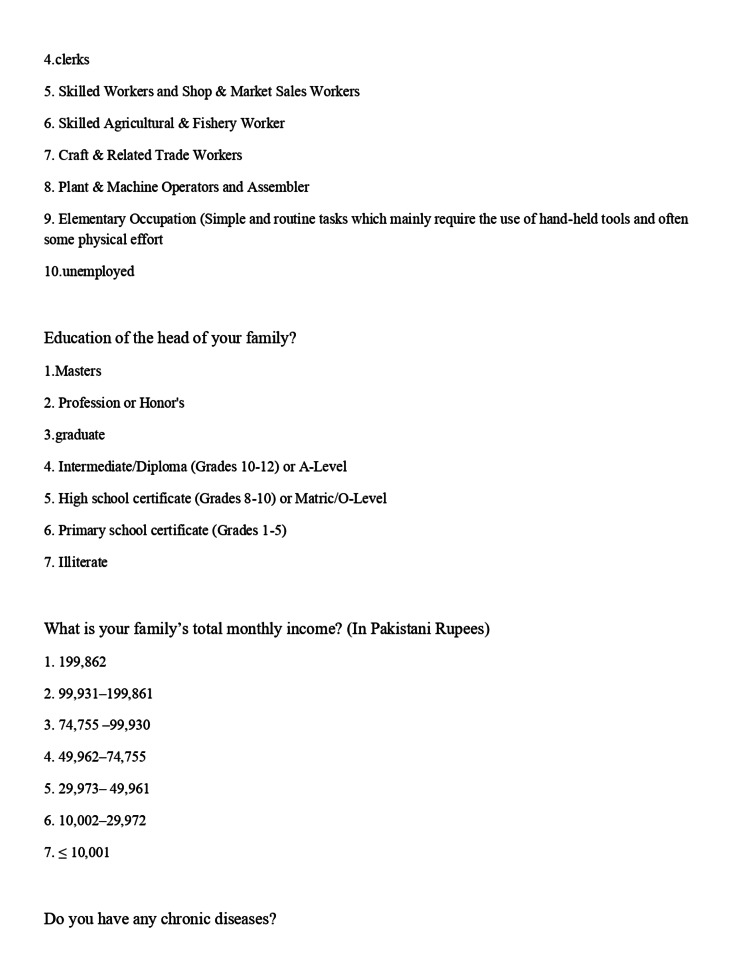
Page 3 of the questionnaire

**Figure 6:**
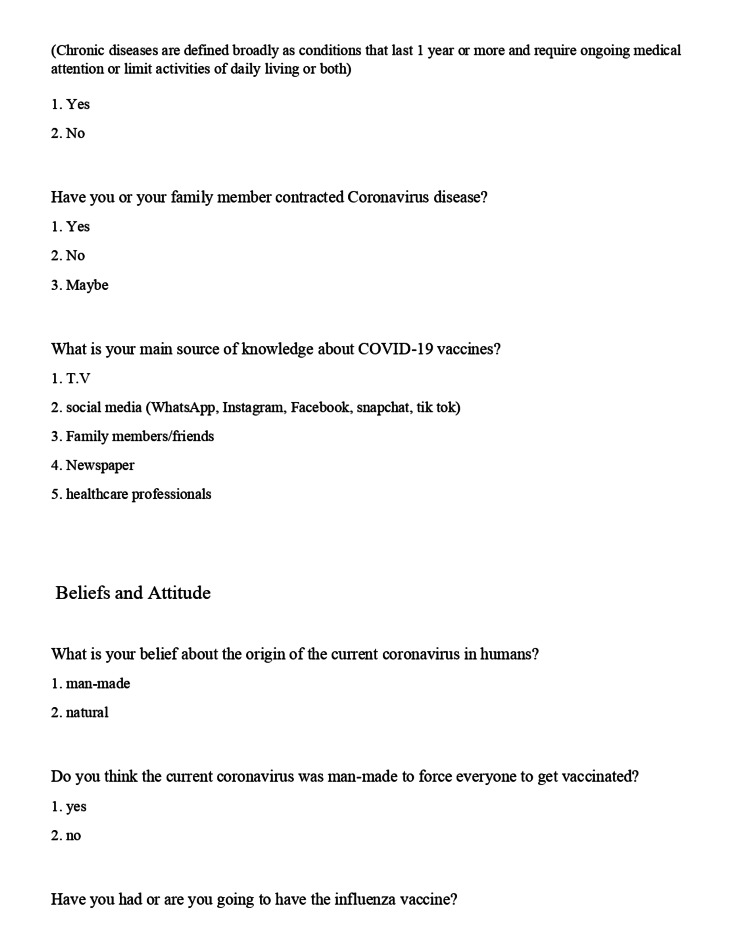
Page 4 of the questionnaire

**Figure 7:**
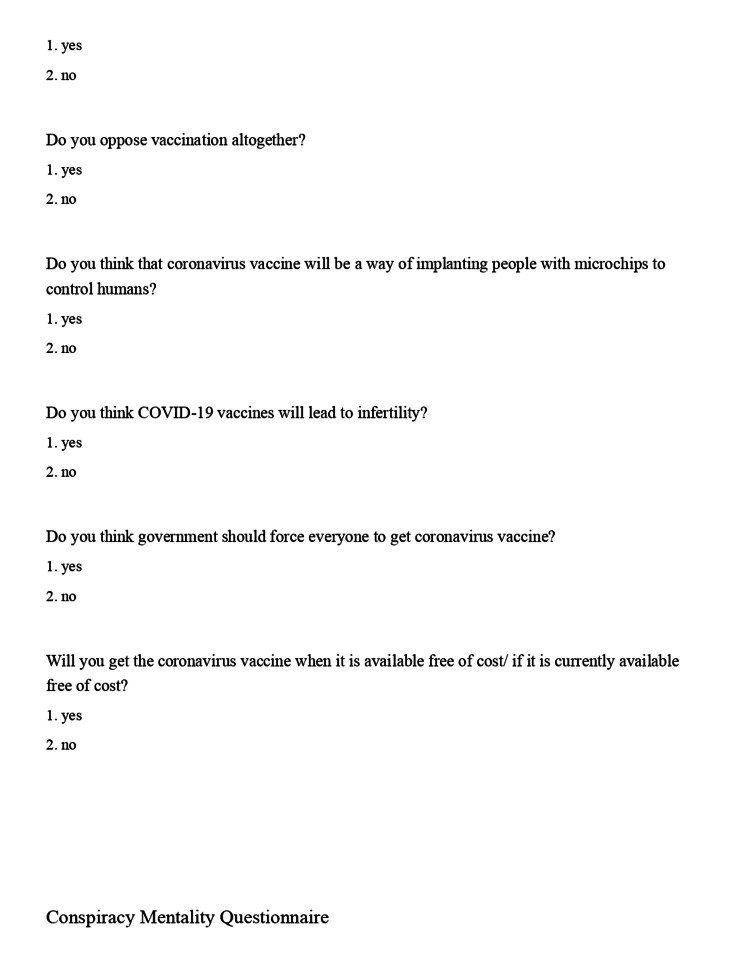
Page 5 of the questionnaire

**Figure 8:**
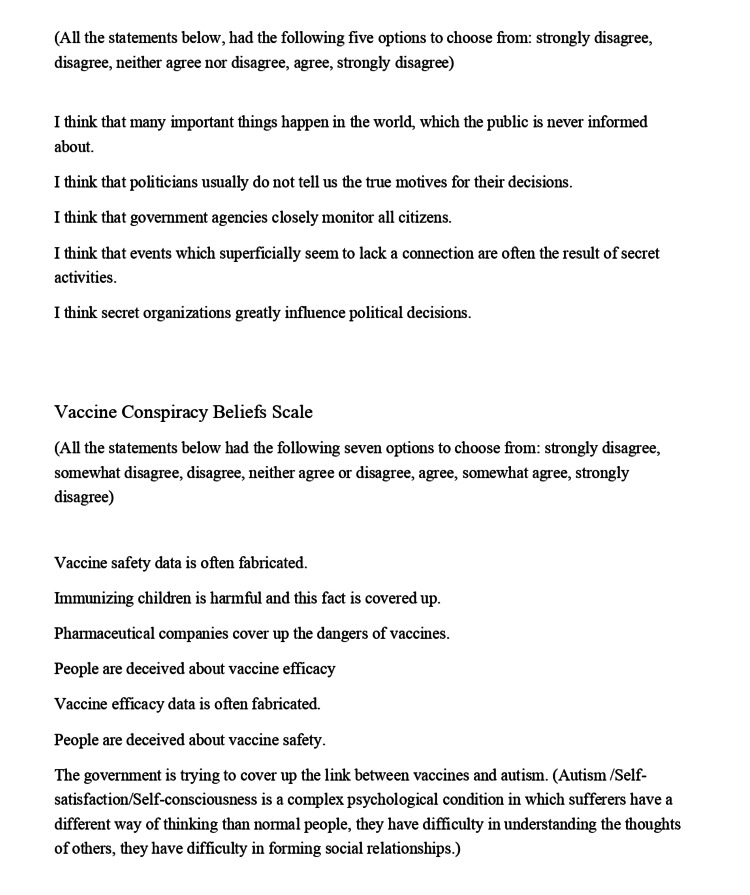
Page 6 of the questionnaire
